# What to Eat in Cold Weather

**Published:** 1890-12

**Authors:** 


### WHAT TO EAT IN COLD WEATHER.

All food contains nitrogen, the substance which supplies muscle, flesh or strength. Carbon, another element contained in food, gives warmth. The colder the weather, the more carbon is required. Alcohol is almost wholly carbon, and, hence, it produces heat, but it does not add a particle of flesh, nor strength. A person feels stronger after taking a drink of spirits, but it is not real strength. It is only strength preternaturally drawn in advance, the nervous system having been stimulated to make that draught, by the influence which the alcohol had on it.

The following substances have a large percentage of carbon, and also a good percentage of nitrogen, so that they furnish warmth in cold weather, and also make muscle :

| | Carbon. | Nitrogen. | |
|---|---|---|---|
| Potatoes, | 11 | 0.36 | Good for heat, not for strength. |
| Milk, | 10 | 0.03 | " " " |
| Butter, | 65 | 0.00 | " " " |
| Lard, | 80 | 0.00 | " " " |
| Soup, | 75 | 0.75 | " " " |
| Wheat, | 39 | 2.00 | " " " |
| Rye, | 38 | 1.00 | " " " |
| Beans, | 88 | 38 | Good for both heat and strength. |
| Roast Beef, | 58 | 15 | " " " |
| Veal, | 52 | 14 | " " " |
| Lean Meat, | 13 | 15 | Better for strength than heat. |

It will be seen that the best food, at this season, is roast beef, or beans, as both furnish heat and muscle. While potatoes, butter, lard, soup, etc., give warmth, they like alcohol furnish little or no strength. Even bread contains but little nourishment.

